# Development of a fibroblast activation protein-targeted PET/NIR dual-modality probe and its application in head and neck cancer

**DOI:** 10.3389/fbioe.2023.1291824

**Published:** 2023-11-03

**Authors:** Danni Li, Xuran Li, Jiaojiao Li, Yanhong Wang, Fei Tan, Xiao Li

**Affiliations:** ^1^ Department of ORL-HNS, Shanghai Fourth People’s Hospital, School of Medicine, Tongji University, Shanghai, China; ^2^ The Royal College of Surgeons in Ireland, Dublin, Ireland; ^3^ The Royal College of Surgeons of England, London, United Kingdom; ^4^ Shanghai Institute of Applied Physics, Chinese Academy of Sciences, Shanghai, China

**Keywords:** fibroblast activation protein, dual-modality imaging, ICG, PET, head and neck cancer, optical imaging

## Abstract

**Purpose:** The combination of near-infrared (NIR) and positron emission tomography (PET) imaging presents an opportunity to utilize the benefits of dual-modality imaging for tumor visualization. Based on the observation that fibroblast activation protein (FAP) is upregulated in cancer-associated fibroblasts (CAFs) infiltrating all solid tumors, including head and neck squamous cell carcinoma (HNSCC), we developed the novel PET/NIR probe [^68^Ga]Ga-FAP-2286-ICG. Preclinically, the specificity, biodistribution and diagnostic properties were evaluated.

**Methods:** Cell uptake assays were completed with the U87MG cell to evaluate the specificity of the [^68^Ga]Ga-FAP-2286-ICG. The tumor-targeting efficiency, biodistribution and optimal imaging time window of the [^68^Ga]Ga-FAP-2286-ICG were studied in mice bearing U87MG xenografts. HNSCC tumor-bearing mice were used to evaluate the feasibility of [^68^Ga]Ga-FAP-2286-ICG for tumor localization and guided surgical resection of HNSCC tumors.

**Results:** The *in vitro* experiments confirmed that [^68^Ga]Ga-FAP-2286-ICG showed good stability, specific targeting of the probe to FAP, and the durable retention effect in high-expressing FAP tumors U87MG cell. Good imaging properties such as good tumor uptake, high tumor-to-background ratios (5.44 ± 0.74) and specificity, and tumor contouring were confirmed in studies with mice bearing the U87MG xenograft. PET/CT imaging of the probe in head and neck cancer-bearing mice demonstrated specific uptake of the probe in the tumor with a clear background. Fluorescence imaging further validated the value of the probe in guiding surgical resection and achieving precise removal of the tumor and residual lesions.

**Conclusion:** In a preclinical model, these attractive [^68^Ga]Ga-FAP-2286-ICG PET/NIR imaging acquired in head and neck cancer make it a promising FAP-targeted multimodal probe for clinical translation.

## 1 Introduction

Head and neck cancer (HNC) is the seventh most common cancer globally. Squamous cell carcinoma, the most common HNC histology for nearly 90% of HNC malignancies, is among the most lethal cancers, with a bleak 40%–50% 5-year survival rate (Bray et al., 2018; Chow, 2020). Currently, the mainstay of treatment for head and neck squamous cell carcinoma (HNSCC) is curative surgery followed by adjuvant treatment, including, but not limited to, radiotherapy, chemotherapy, targeted therapy, and immunotherapy. Early diagnosis and effective treatment are crucial to improve the prognosis of HNSCC patients.

Early detection remains the primary strategy for enhancing patient outcomes in HNSCC. Preoperative imaging techniques, including computed tomography (CT), magnetic resonance imaging (MRI), and positron emission tomography (PET), provide valuable anatomical and metabolic information about the tumor and serve as a reference for surgical resection. Among these imaging modalities, molecular imaging based on nuclear medicine offers high sensitivity for the simultaneous detection and evaluation of multiple lesions, enabling repetitive and non-invasive assessment. A commonly utilized approach involves combining fluorine-18 fluorodeoxyglucose ([^18^F]F-FDG) PET scan with CT or MRI, which has been extensively employed for HNSCC diagnosis, tumor staging, restaging, recurrence detection, and treatment response monitoring (Castaldi et al., 2013). However, the specificity of FDG is insufficient and frequently leads to false positive results, particularly due to inflammatory tissues (Liu, 2019). To enhance the specificity of PET in HNSCC detection, it is crucial to identify the specific molecular profile of HNSCC and develop imaging agents with high affinity for the selected molecules. Numerous HNSCC biomarkers, such as epidermal growth factor receptor (EGFR), fibroblast activation protein (FAP) and integrins, have been identified in this context. These biomarkers primarily located in tumor cells, within the tumor microenvironment and involved in tumor angiogenesis (Li et al., 2022). Promising nuclear medicine imaging agents targeting these relevant biomarkers (e.g., somatostatin receptor, FAP) have been developed and demonstrated superior sensitivity and specificity compared to [^18^F]F-FDG PET in various clinical scenarios (Unterrainer et al., 2018; Chen et al., 2020; Linz et al., 2021).

Surgical resection serves as the primary curative treatment for HNC. Once the HNC is diagnosed by imaging, the head and neck surgeon face the next challenge of defining tumor-free resection margins intraoperatively. Current methods to determine the interface between malignant and normal tissue are largely based on visual inspection and palpation by the surgeon. Notably, this method heavily relies on the expertise of clinician. It is insensitive to finding small residual tumors or metastases, resulting in a positive margin rate for primary HNC resection remaining around 15%–30%, rendering tumor recurrence and reduced patient survival (Orosco et al., 2018; Kain et al., 2020). As surgical techniques and skills have advanced, precision surgery gradually emerged as a distinguished hallmark of modern surgery and became an important part of precision medicine. The fluorescent image-guided surgery (FGS) technique enables intraoperative tumor visualization through fluorescent detector perfectly aligned with the concept of precision surgery. Enhanced permeability and retention (EPR)-based indocyanine green (ICG), antibodies and peptides-guided tracer that bind to tumor cells have been widely employed in developing tumor-targeting fluorescent probes, offering a promising solution for visualizing HNSCC tumors (Rosenthal et al., 2015; Pan et al., 2020; Vonk et al., 2022). Currently, fluorescence imaging (FI) has been the leading modality for combining high sensitivity and spatial resolution. Nonetheless, FI encounters limitations due to tissue penetration. Conversely, nuclear imaging techniques offer unlimited tissue penetration depth, remarkable sensitivity, and quantitative capacity (Zhao et al., 2018). Consequently, a targeted dual-modality probe is appealing because it bridges these two distinct but complementary imaging modalities to improve intraoperative imaging-guided surgery. Recently, ICG has been explored for the development of tumor-targeted PET/NIR dual-modality imaging probes, taking advantage of its optical properties and safety profile. For example, Panikar et al. developed a^89^Zr and ICG dual-labeled anti-caveolin-1 antibody for PET/NIR imaging of caveolin-1 expression in gastric tumors (Surendra Panikar et al., 2023). Additionally, several studies have reported ICG-based PET/NIR probes targeting HER2 and folate receptor for cancer diagnosis and image-guided surgery (Shi et al., 2022; Cao et al., 2023). These emerging studies demonstrate the potential of using ICG to design PET/NIR dual-modality probes for improving cancer detection and surgical guidance.

Using targets that are widely expressed in solid tumors could facilitate the development of more precise approaches to cancer diagnosis and treatment. FAP has emerged as a promising target for diagnosing and treating various malignant tumors (Loktev et al., 2018). The significant overexpression of FAP in cancer-associated fibroblasts (CAFs) in more than 90% of epithelial tumors, including HNCs, while being scarcely detectable in healthy adult tissues, has led to the development of PET imaging using radiolabeled FAP inhibitors (FAPIs) in the field of nuclear medicine (Hamson et al., 2014; Li et al., 2022). Compared to [^18^F]F-FDG, FAP imaging shows good contrast and greater tumor uptake and has very low uptake in the healthy oral cavity, laryngeal mucosa, and brain. This characteristic enhances the diagnostic efficacy of primary HNCs foci and brain metastases (Chen et al., 2020). Among there FAP-binding peptide, FAP-2286, a cyclic peptide-based compound, has been developed with specific binding motifs, displaying remarkable selectivity and a strong affinity for FAP. Due to favorable pharmacokinetic properties, in particular, their rapid clearance from the blood pool accompanied by high tumor-background contrast at early time points and prolonged tumor retention (Baum et al., 2021; Millul et al., 2023). These characteristics provide an optimal basis for designing dual-mode probes. Additionally, a recent study demonstrated the diagnostic precision of [^68^Ga]Ga-FAP-2286 in various types of cancer, including HNC. These findings highlight the potential of [^68^Ga]Ga-FAP-2286 as a safe and effective tool for cancer diagnosis, staging, and restaging, thereby significantly impacting treatment options (Pang et al., 2023).

In this study, PET/NIR dual-modality imaging probe, [^68^Ga]Ga-FAP-2286-ICG, was designed by conjugating FAP-2286 with ICG for NIR fluorescence imaging and DOTA for radiolabeling with ^68^Ga. As a preliminary step towards clinical application, this study explores the feasibility of accurate diagnosis and intraoperative resection of HNC tumor lesions using the new imaging agent [^68^Ga]Ga-FAP-2286-ICG.

## 2 Materials and Methods

### 2.1 Reagents

The synthesis of FAP-2286-ICG precursor was entrusted to Nanchang Tanzhen Bio Co., Ltd. FAP-2286 was purchased from Shanghai Nice-labeling Bio-Technology Co., Ltd. Reagents, including phosphate buffered saline (PBS), cell culture medium, fetal bovine serum (FBS), and trypsin for cell culture were purchased from Gibco (Thermo Fisher Scientific). The Matrigel was purchased from Becton, Dickinson, and Company. [^18^F]F-FDG was purchased from Shanghai Atom Kexing Pharmaceutical Co., Ltd.

### 2.2 Radiopharmaceuticals and quality control

The ^68^Ge/^68^Ga generator (ITG, Germany) was used as the ^68^Ga source, eluted using a 0.05 M HCl solution. FAP-2286-ICG was reconstituted in sodium acetate before adding to the eluted ^68^Ga. The ^68^Ga-labeling was performed by heating a solution containing 4 mL of ^68^GaCl_3_ (111–148 MBq in 0.05 M HCl), 1 mL of 0.25 M sodium acetate, and 30 μg of FAP-2286-ICG precursor at 80°C for 15 min. The radiolabeled ligands had a radiochemical yield of 95% and could thus be used without further purification.

The radiochemical purity (RCP) of [^68^Ga]Ga-FAP-2286-ICG was assessed using radio instant thin-layer chromatography (Radio-iTLC). Glass microfiber chromatography paper impregnated with a silica gel served as the stationary phase and methanol and 0.25 M sodium acetate (1:1, v/v) as the mobile phase.

The *in vitro* stability of [^68^Ga]Ga-FAP-2286-ICG was assessed by co-culture with 0.01 M PBS and FBS at a concentration of 10% (w/w), respectively, and the mixtures were incubated at 37°C for 0.5, 1, and 2 h.

The octanol-water partition coefficient of [^68^Ga]Ga-FAP-2286-ICG was detected by mixing 10 μL of freshly prepared [^68^Ga]Ga-FAP-2286-ICG with 490 μL of PBS (0.1 M, pH 7.4) and 500 μL of n-octanol in a centrifuge tube. The mixture was vortexed for 3 min and centrifuged at 3,000 × rpm for 5 min for layer separation. An aliquot of the aqueous and n-octanol layers was separately collected and measured by *γ* counter (GC-2101, PET Co., Ltd). Log *p* values were calculated using the formula: log *P* = log(total counts in octanol/total counts in PBS).

### 2.3 Optical properties of FAP-2286-ICG

The ultraviolet absorption spectra of FAP-2286-ICG were collected using a UV/Vis spectrophotometer (DU-730, BeckMan Coulter). Fluorescence spectra were collected using a fluorescence spectrometer (FLS980, Edinburgh instruments). FAP-2286-ICG and ICG were dissolved in DMSO at a concentration of 1 mM before being diluted with PBS to a final concentration of 10 μΜ, respectively. The absorbance spectra were taken between 600 and 1100 nm. The emission spectra were recorded in the 790–860 nm range at an excitation wavelength of 770 nm. The concentration dependence of fluorescence intensity of FAP-2286-ICG was assessed with different concentrations of FAP-2286-ICG (0–100 μM). The fluorescent images were collected using a small animal fluorescence imaging system (VISQUE *InVivo* Smart-LF, Vieworks Co., Ltd) and analyzed using CleVue™ software (Vieworks Co., Ltd).

### 2.4 Cell culture, *in vitro* cellular uptake, and cytotoxicity studies

The human glioblastoma-derived cancer cell line U87MG was purchased from EK-Bioscience Biotechnology Co., Ltd. HNSCC cell line, FaDu, CAL27, and CNE2 were purchased from ATCC. Cells were cultured in DMEM or RPMI 1640 medium, supplemented with 10% FBS at 37°C with 5% CO_2_. The immunofluorescence technique was performed to examine FAP expression in U87MG cells. For immunofluorescence, after overnight incubation with anti-FAP primary antibody (1:100; ab53066, Abcam) at 4°C and incubated with FITC-conjugated secondary antibody for 1 h at room temperature. U MG cells were counterstained with DAPI to visualize the nuclei. The fluorescence image was obtained using the Olympus IX-73 fluorescence microscope (Olympus, Tokyo, Japan).

For the cellular uptake study, U87MG cells (2×10^5^ cells/well) were seeded in 24-well plates and incubated at 37°C for 24 h before the experiments (five replicate wells per group). After removing the medium and washing the cells with fresh medium, the cells in each well were treated with DMEM medium containing 0.74 MBq [^68^Ga]Ga-FAP-2286-ICG at 37°C for 30, 60, and 120 min. After removing the medium, the cells were washed twice with cold PBS, lysed with NaOH (1 M) twice (0.5 mL/well), and collected. The bound activity was measured with a *γ*-counter. For the blocking study, a 100-fold excess of the non-radiolabeled precursor was added before treating the cells with [^68^Ga]Ga-FAP-2286-ICG. Subsequent experimental procedure and condition were the same as the aforementioned cellular uptake assay. To further verify whether the introduction of ICG affected the targeting of the precursor, [^68^Ga]Ga-FAP-2286 was incubated with U87MG cells for 2 h as a control group. Additionally, the retention effect of probe on the tumor was verified. Briefly, U87MG cells were incubated with DMEM medium containing approximately 3 μM (based on the content of ICG) FAP-2286-ICG or ICG at 37 °C for 2, 4, 8, and 24 h. After discarding the medium, the cells were washed three times with PBS and imaged using a small animal FI system with ICG mode to quantify the fluorescent signal.

The CCK8 assay was performed to determine the related cytotoxicity of FAP-2286-ICG and [^68^Ga]Ga-FAP-2286-ICG. For the cytotoxicity of FAP-2286-ICG, U87MG cells were incubated with FAP-2286-ICG for 48 h at concentrations ranging from 6.25 to 50 μM. For cytotoxicity of [^68^Ga]Ga-FAP-2286-ICG, U87MG cells were incubated with [^68^Ga]Ga-FAP-2286-ICG for 24 h at radioactive concentrations of 100–1,000 μCi (3.7–37 MBq)/mL.

### 2.5 Establishment of CDX mouse model

All animal studies were performed according to the protocols approved by the Animal Welfare Ethics Committee of Shanghai Fourth People’s Hospital. Six to eight-week-old female BALB/c nude mice were purchased from Beijing Vital River Laboratory Animal Technology Co., Ltd. To establish cell-derived xenograft (CDX) models, the mice were implanted subcutaneously in the flanks with 5 × 10^6^ U87MG cells in PBS containing 50% Matrigel. HNSCC cell lines (FaDu, CAL27, and CNE2) were inoculated subcutaneously on the right flanks of nude mice. The diameter of each tumor was determined and documented every 2–3 days. When the tumor reached 300–400 mm^3^, the mice were used for the imaging study. The U87MG tumor-bearing mice were used to study the *in vivo* targeting, biodistribution, and optimal FI time window of [^68^Ga]Ga-FAP-2286-ICG. HNSCC tumor-bearing mice were used to evaluate the feasibility of [^68^Ga]Ga-FAP-2286-ICG for tumor localization and guided surgical resection of HNSCC tumors.

### 2.6 PET/CT and fluorescence imaging

All imaging acquisition was performed with a clinical PET/CT scanner (Biograph64, Siemens Healthcare, Germany). For [^68^Ga]Ga-FAP-2286-ICG PET/CT, mice were anesthetized with 50 μL 3% (w/w) pentobarbital sodium first, and 10 MBq (1 nmol) [^68^Ga]Ga-FAP-2286-ICG was intravenously injected into tumor-bearing mice. PET/CT scan started with a low dose CT scan and was followed by a PET scan with parameters as follows: for CT, tube voltage, 120 kV; tube current, 35 mA; pitch, 1.0; reconstructed layer thickness, 0.75 mm; for PET, acquisition of whole-body images was finished in a bed with 3 min. The data analysis of PET images was performed with INVEON software (Siemens). Regions of interest (ROIs), including heart, lung, liver, kidney, intestine, brain, muscle, and tumor, were manually drawn to quantify tracer uptake by maximum standard uptake values (SUV_max_). Tumor-to-background ratios (TBRs) were defined as the SUV_max_ of the tumor divided by the SUV_max_ of muscle (i.e., SUV_max_ tumor/SUV_max_ muscle).

FI was performed using a small animal FI system with ICG mode: The NIR filter set (Excitation: 740**–**790 nm; Emission: 810**–**860 nm) was used for ICG fluorescence. The fluorescence intensity was calculated by the mean fluorescence intensity (MFI).

### 2.7 *In vivo* targeting efficiency, biodistribution, optimal dose and fluorescence imaging time window exploration

PET/CT imaging was performed in U87MG tumor-bearing mice to assess the tumor-targeting efficiency and biodistribution of [^68^Ga]Ga-FAP-2286-ICG *in vivo*. The mice were injected with 10 MBq (1 nmol) of [^68^Ga]Ga-FAP-2286-ICG through the tail vein, and images were performed at 30, 60, and 90 min after injection. The blocking group was pre-injected with the 50-fold molar excess of non-radiolabeled precursor 1 h in advance.

[^18^F]F-FDG PET/CT were performed on the same day prior to the [^68^Ga]Ga-FAP-2286-ICG PET/CT scans, with an interval of at least 8 h. The mice were fasting for 8 h before [^18^F]F-FDG scans. [^18^F]F-FDG PET/CT was performed 1 h after injection of 7.4 MBq of [^18^F]F-FDG via the tail vein in mice. The scanning parameters were the same as those of [^68^Ga]Ga-FAP-2286-ICG PET/CT but with a difference in isotope item.

To determine the optimal dose of FAP-2286-ICG for *in vivo* fluorescence imaging, normal nude mice were intravenously injected with 250 μL of FAP-2286-ICG at concentrations ranging from 0 to 25 μM. FI was performed using a small animal imaging system at 10 min post-injection to evaluate the fluorescence signal at each dose. Based on an initial screening that identified detectable fluorescence signals at doses of 6.25–25 μM FAP-2286-ICG, a biodistribution study was performed in normal nude mice at multiple time points after intravenous injection of 6.25, 12.5 or 25 μM FAP-2286-ICG.

To evaluate the optimal imaging time window, NIR fluorescence images of U87MG tumor-bearing mice were collected at different time points (0.5, 1, 2, 24, 48, and 72 h) post-injection of FAP-2286-ICG (3.16 μg, 1.37 nmol). Following *in vivo* FI, mice were euthanized, and their xenografts and major organs were harvested for *in vitro* FI analysis. The ROIs were drawn manually in the tumor and organs on the fluorescence image to account for the distribution of FAP-2286-ICG in the tissue. MFI levels from organs and the TBRs using tumor/muscle values were evaluated.

### 2.8 PET/NIR imaging in HNSCC tumor-bearing mice

HNSCC tumor-bearing mice underwent PET/CT imaging 1 h after intravenous tail injection of 10 MBq (approximately 1.37 nmol) of [^68^Ga]Ga-FAP-2286-ICG to localize the tumor. Subsequently, FI was performed 72 h later to guide surgical resection. After removing the fluorescent tissue, FI was performed again to detect residual fluorescent tissue. The residual fluorescent tissue was then removed. FI and resection were repeated until no fluorescence was observed on the tumor margin.

### 2.9 Toxicity study of [^68^Ga]Ga-FAP-2286-ICG

The BALB/c mice were sacrificed and collected from the heart, liver, spleen, lung, and kidney to determine toxicity with hematoxylin and eosin (H&E) staining. The serum was collected to measure the levels of aspartate aminotransferase (AST), alanine aminotransferase (ALT), and blood urea nitrogen (UREA). Body weight was recorded every 5 days up to 20 days postinjection. The mice with intravenous injections of saline served as a control group.

### 2.10 H&E staining and immunohistochemistry

First, the tumor tissues were harvested and fixed in formalin. After being embedded in paraffin, the samples were subsequently sectioned to a thickness of 4 μm and stained with H&E and FAP immunohistochemistry (IHC) according to the standard protocol. The following primary antibodies were used: anti-FAP mAb (1:100, ab53066, Abcam, Shanghai, China). ImageJ software and the IHC Profiler plugin were applied to assess IHC slides qualitatively.

### 2.11 Statistical analysis

All the data in this study were given by mean ± SD. The Student’s t-test was used to compare the differences between the two groups. A *p*-value of 0.05 or less was considered statistically significant. Graph preparation and statistics were performed using GraphPad Prism version 9.3.

## 3 Results

### 3.1 Optical properties of FAP-2286-ICG

**FIGURE 1:**
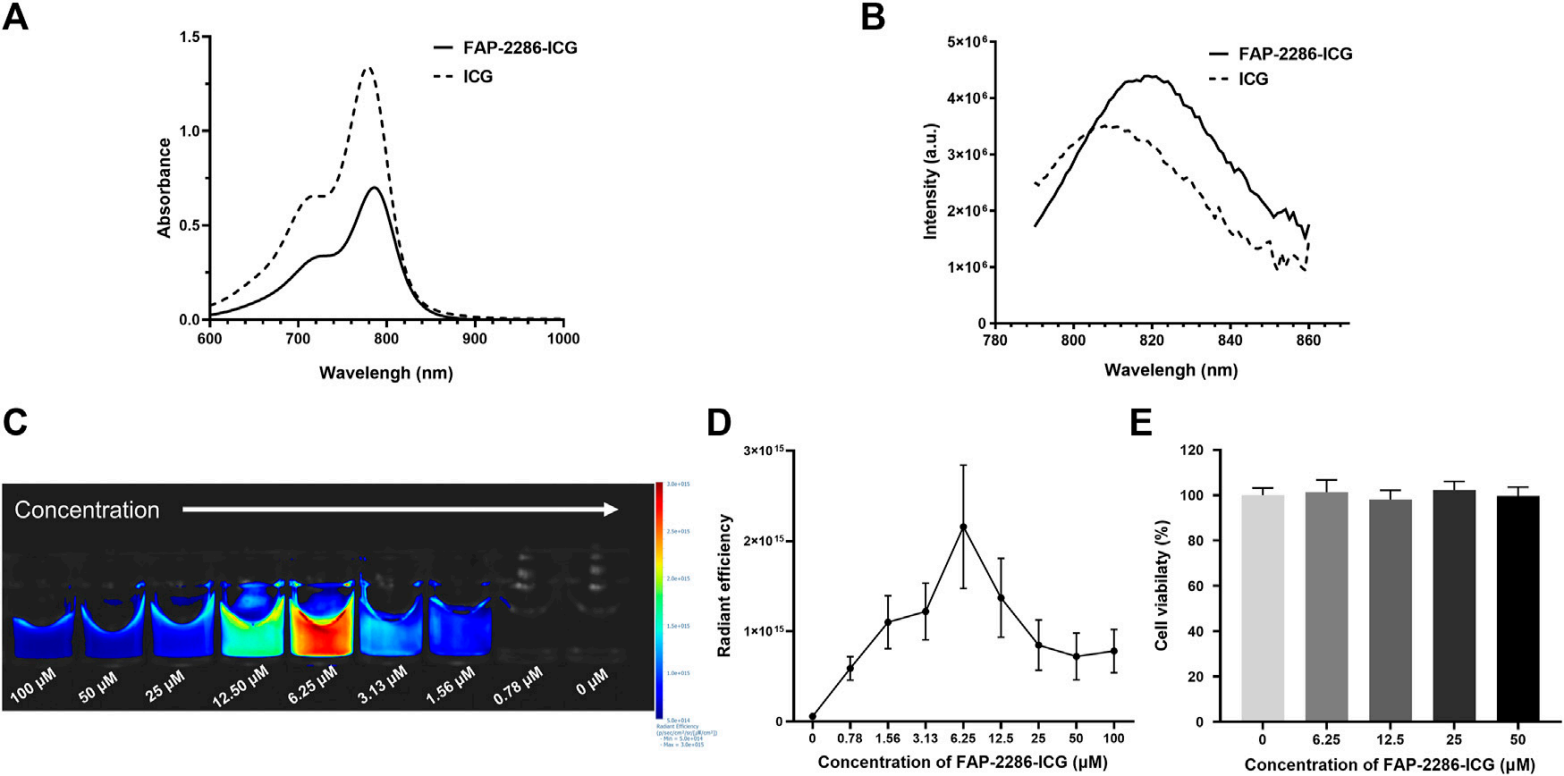
Optical properties of FAP-2286-ICG. **(A)** Absorbance spectra of FAP-2286-ICG and free ICG; **(B)** Fluorescence emission spectra of FAP-2286-ICG and free ICG; Visual **(C)** and quantitative **(D)** results of the relationship between FAP-2286-ICG concentration and relative fluorescence intensity *in vitro*; **(E)** Viability of U87MG cells incubated with different concentrations of FAP-2286-ICG for 48 h. Data were expressed as mean ± SD (*n* = 3).

The mass spectrometry data indicated that FAP-2286-ICG was successfully synthesized with a molecular weight of 2,312.91 Da (Supplementary Figure S1). Furthermore, the chemical purity of FAP-2286-ICG was over 99%, as determined by HPLC (Supplementary Figure S2). The optical properties of FAP-2286-ICG were used to assess the migration of absorption and fluorescence emission peaks induced by structural modifications. The absorption spectra of FAP-2286-ICG and ICG were basically the same, with absorption peaks at 786 and 779 nm, respectively (Figure 1A). FAP-2286-ICG exhibited NIR-I emission peaked at around 819 nm, slightly higher than that of ICG (808 nm) (Figure 1B). As shown in Figures 1C, D, the fluorescence intensity of FAP-2286-ICG was slightly increased at low concentrations (0–6.25 μM), whereas it was decreased at high concentrations (6.25–100 μM). A clear dose-dependent trend of increasing fluorescence intensity was observed when the concentration range of FAP-2286-ICG was 0–6.25 μM (*R* ^2^ = 0.9305, *p* = 0.0079). At a FAP-2286-ICG concentration greater than 6.25 μM, a distinct self-quenching phenomenon is observed. Additionally, no cytotoxicity was detected for FAP-2286-ICG at concentrations up to 50 μM (Figure 1E).

### 3.2 Radiopharmaceutical characteristics of [^68^Ga]Ga-FAP-2286-ICG

**FIGURE 2:**
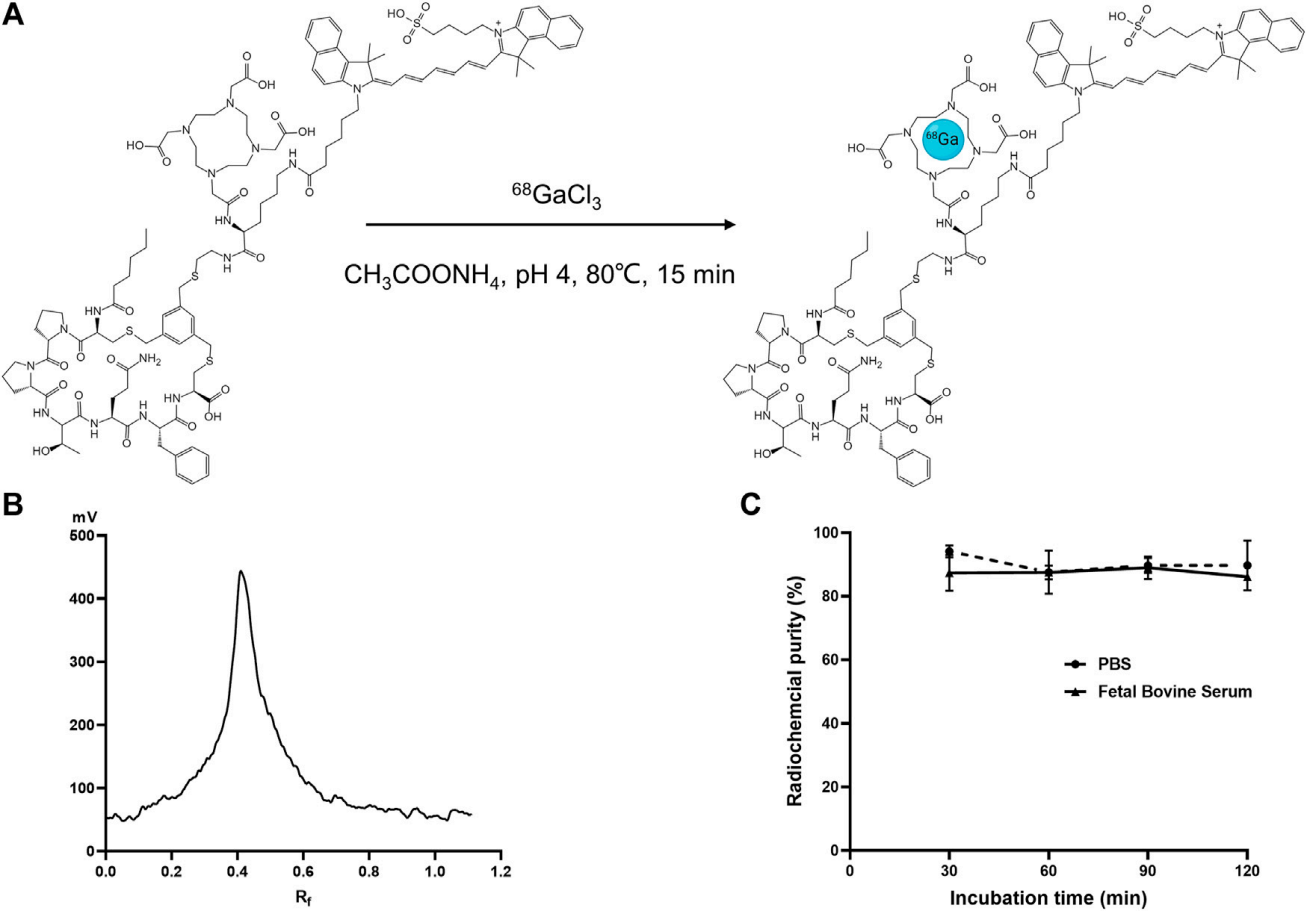
**(A)** Schematic presentation of labeling synthesis of [^68^Ga]Ga-FAP-2286-ICG; **(B)** Radioactive TLC spectrum of ^68^Ga-labeled FAP-2286-ICG; **(C)** The *in vitro* stability of [^68^Ga]Ga-FAP-2286-ICG in 0.01 M PBS and fetal bovine serum concentration of 10% (w/w) at 37°C.

Figure 2A illustrates the labeling process of [^68^Ga]Ga-FAP-2286-ICG. [^68^Ga]Ga-FAP-2286-ICG was successfully prepared by a simple one-step reaction (pH = 4, 15 min) at 80°C without further purification with a labeling rate reached >95% (Figure 2B). The radioligands were used for *in vitro* and *in vivo* experiments without further purification. Figure 2C presents that the RCP of [^68^Ga]Ga-FAP-2286-ICG was greater than 85% after incubation in PBS and 10% FBS solution at 37°C for 2 h, as revealed by radio-iTLC, showing good stability *in vitro.* The lipid-water distribution of [^68^Ga]Ga-FAP-2286-ICG showed that the majority of radioactivity was concentrated in the water phase, and the partition coefficient of [^68^Ga]Ga-FAP-2286-ICG was calculated as log *p* = −1.20 ± 0.18, indicating the favorable hydrophilicity.

### 3.3 *In vitro* cellular uptake and cytotoxicity studies

**FIGURE 3:**
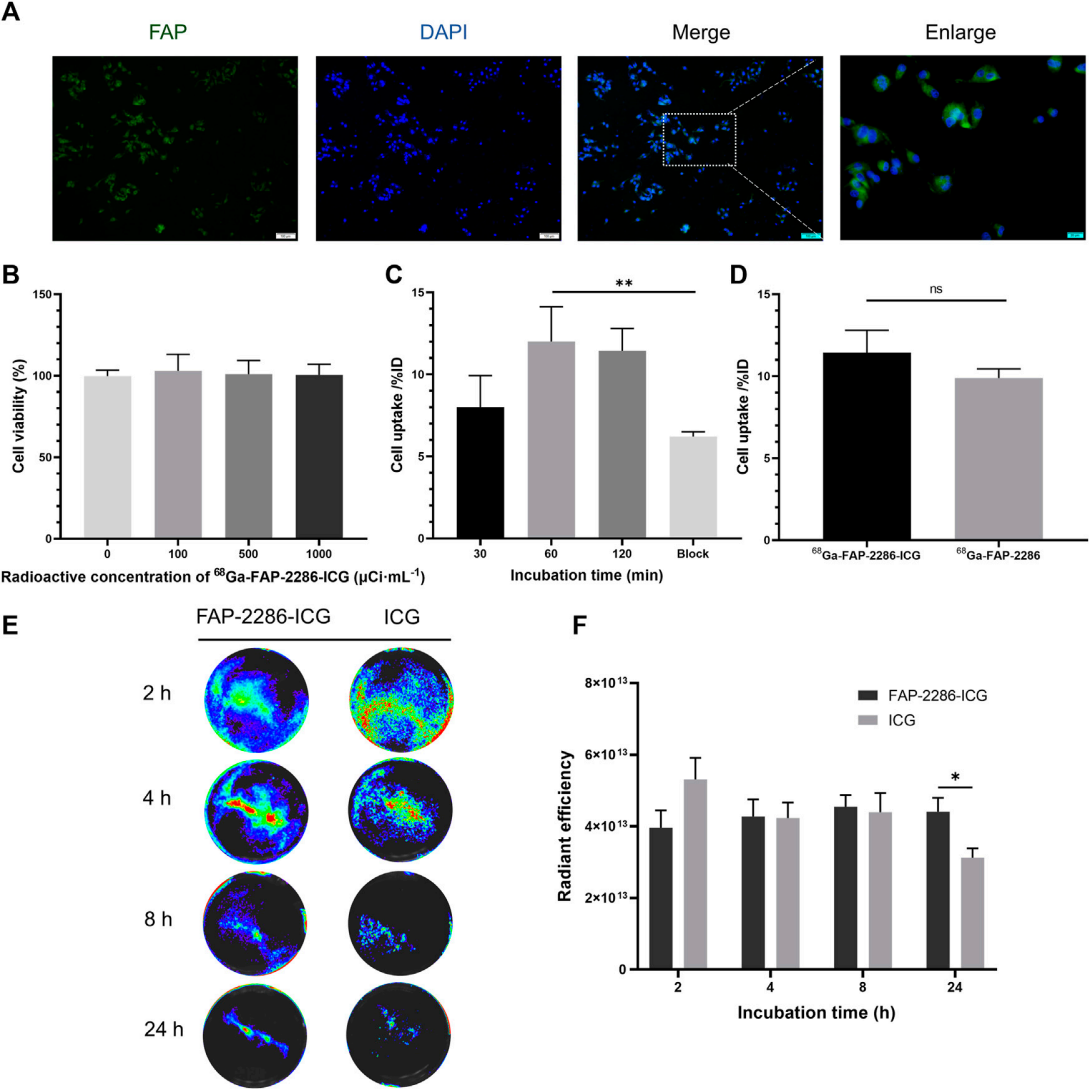
**(A)** Representative immunofluorescence staining of FAP protein in U87MG cell. FAP was indicated by positive staining (green), and nuclear condensation was indicated by DAPI nuclear staining (blue). Scale bar: 100 μm; **(B)** Viability of U87MG cells incubated with the different radioactive concentrations of [^68^Ga]Ga-FAP-2286-ICG for 24 h (n = 3); **(C)** The radioactivity uptake of [^68^Ga]Ga-FAP-2286-ICG in U87MG cells; **(D)** Cellular uptake of [^68^Ga]Ga-FAP-2286-ICG compared to [^68^Ga]Ga-FAP-2286 in U87MG cells; Representative optical image **(E)** and quantification **(F)** of U87MG cells interaction after incubation for 2, 4, 8, and 24 h with 3 μM of either FAP-2286-ICG or ICG (*n* = 5). ns: not statistically significant; *p < 0.05; **p < 0.01.

Before *in vitro* and *in vivo* evaluation of [^68^Ga]Ga-FAP-2286-ICG, the expression of FAP in U87MG cells was assessed by immunofluorescence staining. Figure 3A shows that the surface of U87MG cells displayed homogeneous and high levels of FAP expression. For the cytotoxicity experiments, even when the radioactive concentration of [^68^Ga]Ga-FAP-2286-ICG reached 37 MBq/mL, almost no cytotoxicity was observed, and the cell viability of U87MG cells still reached 100% (Figure 3B). Figure 3C shows that for the cellular uptake assay, in FAP-positive U87MG cells, with an increase in incubation time, cellular uptake of [^68^Ga]Ga-FAP-2286-ICG gradually increased, indicating that this property is time-dependent. The highest uptake of [^68^Ga]Ga-FAP-2286-ICG was observed at 60 min (12.00% ± 1.73%ID), which could be blocked to 6.88% ± 0.37%ID by the addition of excess non-radiolabeled precursor (*p = 0.0094*), indicating the specific binding between [^68^Ga]Ga-FAP-2286-ICG and FAP. Additionally, there was no statistically significant difference between the cellular uptake of [^68^Ga]Ga-FAP-2286-ICG and that of [^68^Ga]Ga-FAP-2286 (*p* = 0.1945) (Figure 3D). These data suggested that the introduction of ICG did not affect the FAP targeting.

To verify the retention effect of the probe in tumor, FI assessed the uptake of FAP-2286-ICG and ICG by U87MG cells. Figures 3E, F show that FAP-2286-ICG exhibited more efficient retention capabilities after incubation with U87MG cells for 24 h than those of ICG ([4.40×10^13^ ± 3.39×10^12^ p/sec/cm^2^/sr/[μW/cm^2^]] vs [3.13×10^13^ ± 2.21×10^12^ p/sec/cm^2^/sr/[μW/cm^2^]], *p* = 0.0235), possibly due to the FAP-2286-mediated retention of the probe. These results suggested the efficient cellular uptake and retention of FAP-2286-ICG, the basis for the optimal *in vivo* imaging effect.

### 3.4 PET imaging of U87MG tumor-bearing mice

**FIGURE 4:**
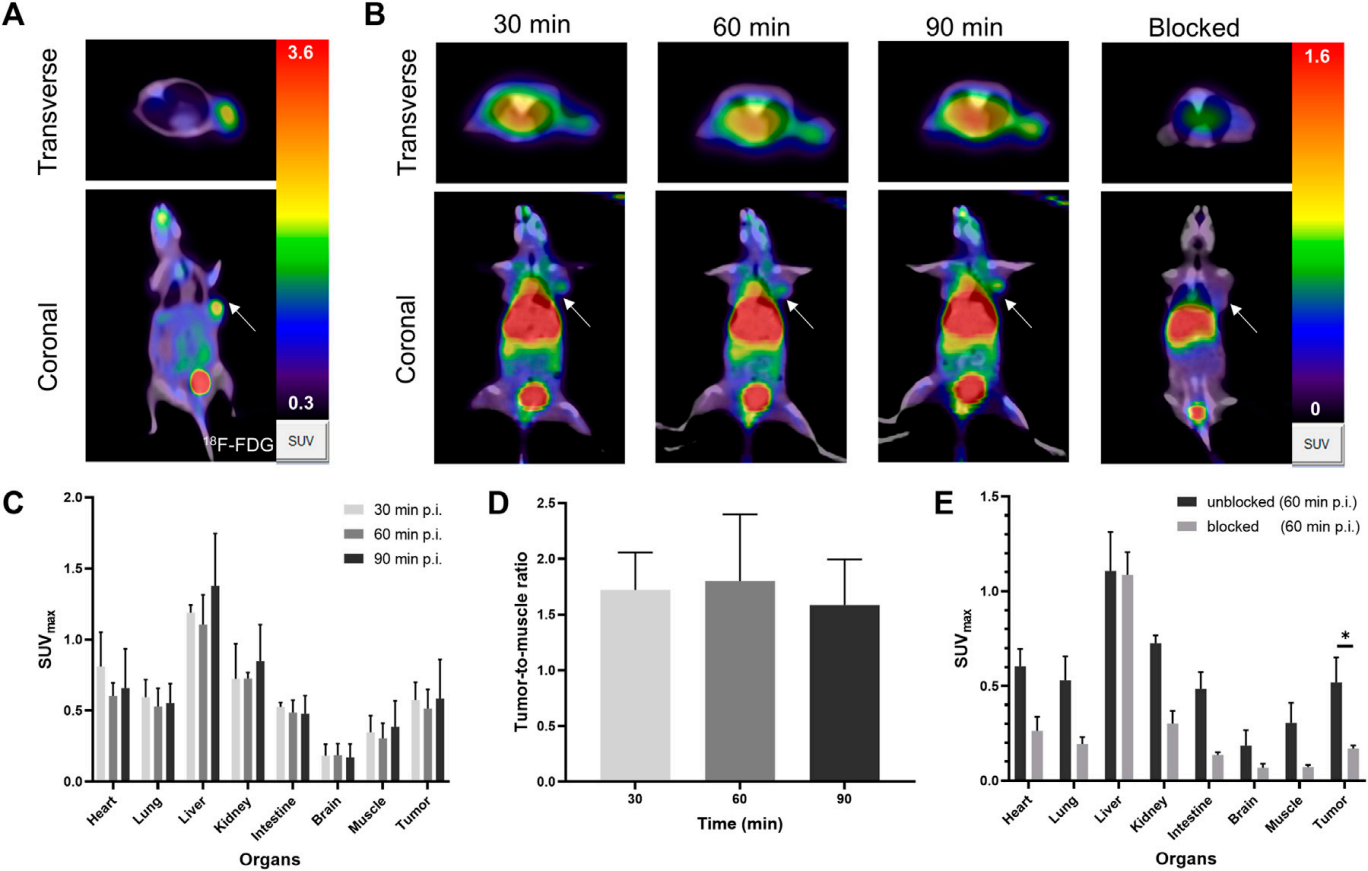
**(A)** The PET/CT of [^18^F]F-FDG in U87MG tumor-bearing mice at 60 min post-injection. **(B)** Representative PET/CT imaging of [^68^Ga]Ga-FAP-2286-ICG at 30, 60, 90 min post injection and block with excess unlabeled FAP-2286-ICG at 60 min in U87MG tumor-bearing mice; the white arrow indicates the location of the tumor; **(C)** The SUV_max_ of major organs in U87MG tumor-bearing mice at three time-points (30, 60, and 90 min); **(D)** The dynamic changes of SUV_max_ ratio of tumor-to-background at three time-points (30, 60, and 90 min); **(E)** [^68^Ga]Ga-FAP-2286-ICG uptake in U87MG xenograft mice with unblocking or blocking of FAP by excess non-radiolabeled tracer precursor. Data were expressed as mean ± SD (*n* = 3). *p < 0.05.

Tumors were highly metabolically active by [^18^F]F-FDG PET/CT, with SUV_max_ of 0.36 ± 0.06, indicating an aggressive phenotype (Figure 4A). The *in vivo* distribution and metabolic characteristics of [^68^Ga]Ga-FAP-2286-ICG in U87MG tumor-bearing mice were evaluated in real-time and noninvasively via PET/CT imaging at 30, 60 and 90 min post-injection of the radiotracer. PET/CT imaging showed that radioactivity accumulated mainly in the liver, kidneys and bladder, indicating that [^68^Ga]Ga-FAP-2286-ICG was mainly eliminated through liver metabolism and kidney excretion (Figure 4B). Figures 4B, C show that [^68^Ga]Ga-FAP-2286-ICG were highly uptake by the tumor at 30 min after injection. Furthermore, SUV_max_ of the tumor was 0.57 ± 0.10 at 30 min, 0.51 ± 0.11 at 60 min, and 0.58 ± 0.23 at 90 min, indicating constant uptake of [^68^Ga]Ga-FAP-2286-ICG in the tumor. Other organs demonstrated low nonspecific binding that gradually decreased, resulting in low background signal and favorable tumor-to-background ratios (Figure 4D). The background uptake of the radiotracer in the brain was very low, with an average of 0.18 ± 0.07 at three time points after injection. These results demonstrated good supporting on the utility of this radiotracer for imaging brain tumors.

Regarding the blocking group, a decrease in radioactivity was detected in most organs (Figure 4B). Moreover, the tumor uptake decreased most significantly (SUV_max_, 0.52 ± 0.11 vs 0.17 ± 0.01; 60 min p. i., *p = 0.0106*) (Figure 4E). The results confirmed the specific binding between [^68^Ga]Ga-FAP-2286-ICG and FAP. These results suggested that [^68^Ga]Ga-FAP-2286-ICG had specific FAP targeting properties, resulting in high tumor uptake and good pharmacokinetic properties.

### 3.5 NIR fluorescence imaging using FAP-2286-ICG in U87MG tumor-bearing mice

Based on the visual and quantitative *in vitro* results, and fluorescence dose screening experiments in normal nude mice, 6.25 μM was selected as the optimal dose for subsequent *in vivo* fluorescence imaging. This preserved a sufficient fluorescence signal while enabling quick clearing from background tissues (Supplementary Figure S3).

**FIGURE 5:**
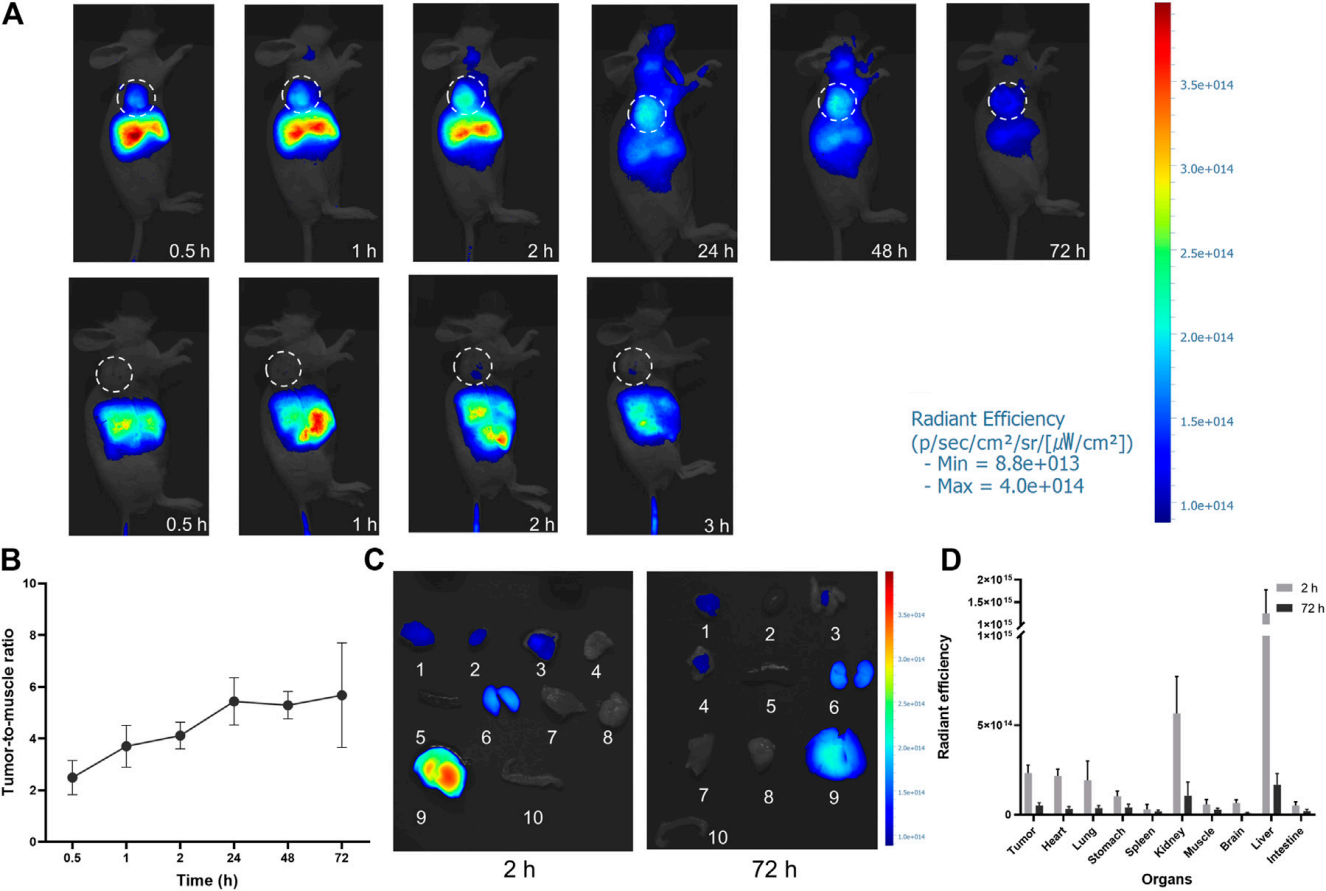
*In vivo* NIR imaging of FAP-2286-ICG. **(A)** NIR images of U87MG tumor-bearing mice at different time points after injection of FAP-2286-ICG (upper) or ICG (bottom) (*n* = 3). The dotted circle indicates the location of the tumor; **(B)** Tumor-to-muscle ratios of FAP-2286-ICG in U87MG tumor-bearing mice at different time points post-injection; **(C)** Biodistribution of FAP-2286-ICG in U87MG tumors and main organs at 2 and 72 h post-injection. 1: tumor, 2: heart, 3: lung, 4: stomach, 5: spleen, 6: kidney, 7: muscle, 8: brain, 9: liver, 10: intestine. **(D)** Quantification of optical signal in main organs at 2 and 72 h post-injection.

U87MG tumor-bearing mice were imaged at several time points (0.5, 1, 2, 24, 48, and 72 h) after FAP-2286-ICG injection to determine the optimal time for FI (Figure 5A, upper panel). FI showed a significant fluorescence contrast in tumors compared to normal tissue as early as 0.5 h post-injection, which was consistent with PET/CT imaging (Figure 4B). The fluorescence intensity at the tumor sites showed a time-dependent increase and reached a maximum signal-to-background ratio at 24 h post-injection and remained at this ratio at 48 and 72 h (Figure 5B). The ratio of tumor-to-muscle, which was initially measured at 2.50 ± 0.54 after 0.5 h, exhibited a significant increase of more than two-fold at 24 h, with a recorded value of 5.44 ± 0.74. Subsequently, the signals emanating from the tumor exhibited a slightly reduction after 24 h, while persisting subsequent to intravenous administration at the 72-h mark. Notably, the tumor tissue had a distinct visual profile within 2 h after injection compared to 24 h after injection. This suggested that the optimal surgical operation time should be performed within the time frame of massive tumor accumulation and clear profile after injection of FAP-2286-ICG. In contrast, the free ICG group presented no fluorescence signals in the tumor site (Figure 5A, bottom panel), demonstrating that FAP-2286-ICG could predominantly improve the tumor-targeting ability and possess longer tumor retention behavior.

To verify the uptake of FAP-2286-ICG in non-invasive FI, major organs and tissues were imaged *in vitro* at time points 2 and 72 h after FAP-2286-ICG injection, and fluorescence was quantified (Figures 5C, D). *In vitro* imaging confirmed a sustained high accumulation level of FAP-2286-ICG in tumor tissue. Additionally, within 2 h post-injection, a significant fluorescence signal could be observed in the tissues of heart, lungs, liver, and kidney. The liver showed the most apparent fluorescent signal. The fluorescence intensity of each organ decreased over 72 h following injection, compared to the initial intensity observed at 2 h. The fluorescence signal of the heart and lungs was almost undetectable at 72 h after injection. The significant fluorescence signal in the liver and kidney indicated that the FAP-2286-ICG was metabolized by the liver and excreted by the kidney.

### 3.6 *In vivo* PET/NIR imaging study of HNSCC tumor-bearing mice

Since the results of NIRF imaging and PET imaging were consistent in xenograft models with high expression of FAP, as proof of the diagnosis and guided surgery concept, this study further explored the possibility of [^68^Ga]Ga-FAP-2286-ICG PET/CT for HNC diagnosis and NIR real-time imaging for guiding HNC surgery *in vivo*. Meanwhile, this study further explored the feasibility of achieving two consecutive imaging modalities (i.e., PET/CT followed by FI) with a single injection, in light of the almost identical molar mass of precursors per mouse injection for PET and FI that had previously been confirmed in experiments, and the prolonged accumulation of FAP-2286-ICG in tumors.

**FIGURE 6:**
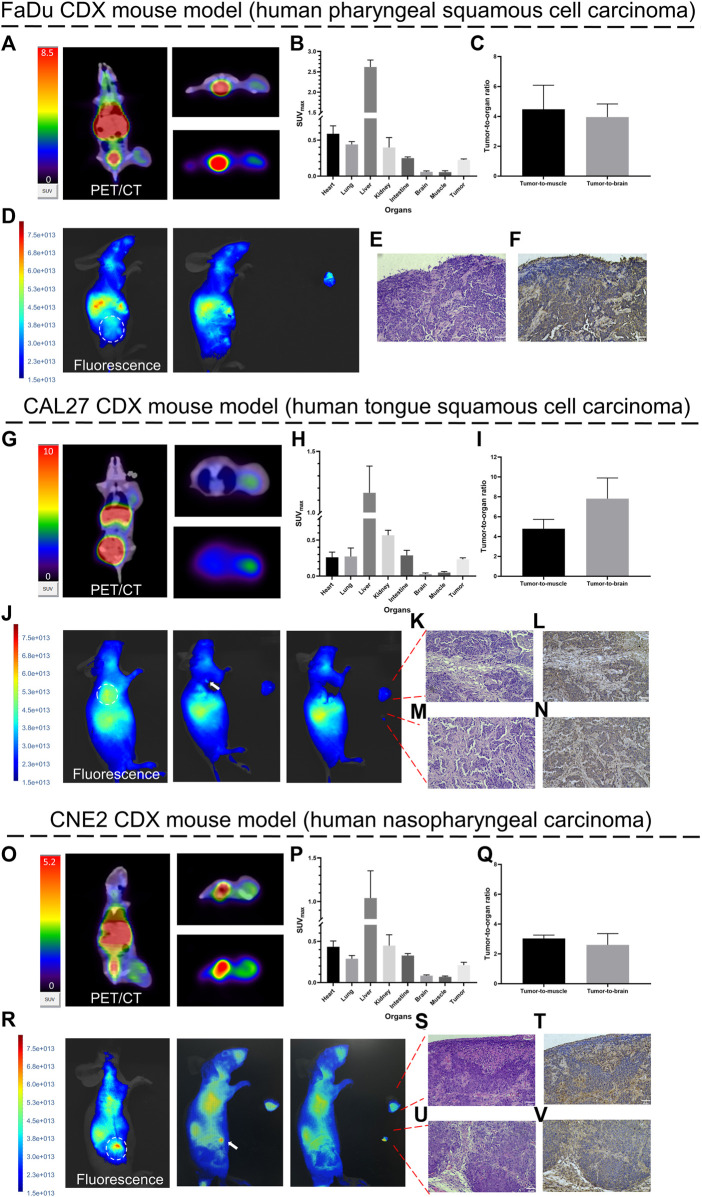
Proof-of-concept of diagnosis and fluorescence-guided surgery studies in HNCs tumor-bearing mice. Representative PET/CT images (1 h post-injection) and fluorescence images (72 h post-injection) of [^68^Ga]Ga-FAP-2286-ICG in FaDu **(A, D)**, CAL27 **(G, J)**, and CNE2 **(O, R)** tumor-bearing mice. White arrows indicate residual fluorescence signal in the surgery bed; SUV_max_ of critical organs in FaDu **(B)**, CAL27 **(H)** and CNE2 **(P)** tumor-bearing mice at 1 h post-injection; Tumor-to-organ ratios of [^68^Ga]Ga-FAP-2286-ICG in FaDu **(C)**, CAL27 **(I)** and CNE2 **(Q)** tumor-bearing mice at 1 h post-injection; pathology and FAP immunohistochemistry of resected tissue of FaDu **(E. F)**, CAL27 **(K–N)** and CNE2 **(S–V)** tumor-bearing mice confirmed the presence and positive staining of FAP in tumor stroma (stained brownish yellow); magnification × 200. Scale bar: 50 μm. Data were expressed as mean ± SD (*n* = 3).

Several representative PET/CT coronal images and the corresponding cross-sectional and maximum intensity projection (MIP) cross-sectional images of all three HNC tumor-bearing mice at 1 h after injection are presented (Figures 6A, G, O). The distribution of [^68^Ga]Ga-FAP-2286-ICG was approximately the same in the three HNC models. [^68^Ga]Ga-FAP-2286-ICG PET/CT displayed significantly high uptake in all three tumor types of HNC. Meanwhile, PET/CT showed an inhomogeneous distribution of radioactivity signals within tumors at relatively high spatial resolution. The quantitative analysis of the SUV_max_ values of each important organ demonstrated that normal tissues, including the brain, lung, intestine, and muscle, had low uptake; The organs with the highest non-target uptake were the kidneys, liver, and heart, which were involved in the tracer blood circulation (Figures 6B, H, P). Tumor-to-muscle and tumor-to-brain ratios, based on SUV_max_, were calculated. Figures 6C, I, Q show the tumor-to-organ (i.e., tumor-to-muscle and tumor-to-brain) ratios of FaDu, CAL27, and CNE2 tumor-bearing mice at 1 h post-injection. A high tumor-to-background signal ratio was observed in the three HNC tumor-bearing mice 1 h after injection. The tumor-to-muscle ratio ranged from 2.78 to 6.20, with a median of 4.10 ± 1.17. The tumor-to-brain ratio ranged from 2.12 to 9.60, with a median of 4.80 ± 2.48. These results demonstrated the tumor-specific uptake of [^68^Ga]Ga-FAP-2286-ICG in HNSCCs, allowing early achievement of a high tumor-to-background ratio and facilitating lesion detection.

Fluorescence images of all three HNC tumor-bearing mice at 72 h after injection of [^68^Ga]Ga-FAP-2286-ICG were generally consistent with the findings on the corresponding PET/CT images. Figures 6D, J, R demonstrates that tumors and normal tissue could be visually distinguished by [^68^Ga]Ga-FAP-2286-ICG FI. The tumor-to-background signal ratio in mice with FaDu, CAL27 and CNE2 tumors at 72 h after injection was favorable, with FaDu tumor-bearing mice having a ratio of 5.95 ± 2.92, CAL27 having a ratio of 2.77 ± 1.70 and CNE2 having a ratio of 5.8 ± 1.15. Notably, a heterogeneous fluorescence signal was observed at the tumor site on both *in vivo* and *in vitro* fluorescence images, which aligned with the heterogeneous radioactive uptake observed on PET/CT.

Similarly, it is worth noting that after the HNCs tumor was removed under white light, small residual foci of fluorescence were detected via FI (Figures 6J, R). The fluorescence intensity in the lesions was significantly higher than that of the surrounding tissue. Histology of the tumor bed and residual foci revealed that tumor cell nuclei were enlarged, with irregular nuclear contours, and displayed clear to hyperchromatic chromatin (Figures 6E, K, M, S, U). A brown-stained region surrounding the tumor cells was revealed by FAP staining, with some exhibiting streak-like structures that indicate fibroblasts expressing FAP (Figures 6F, L, N, T, V).

### 3.7 The toxicity study of [^68^Ga]Ga-FAP-2286-ICG

**FIGURE 7:**
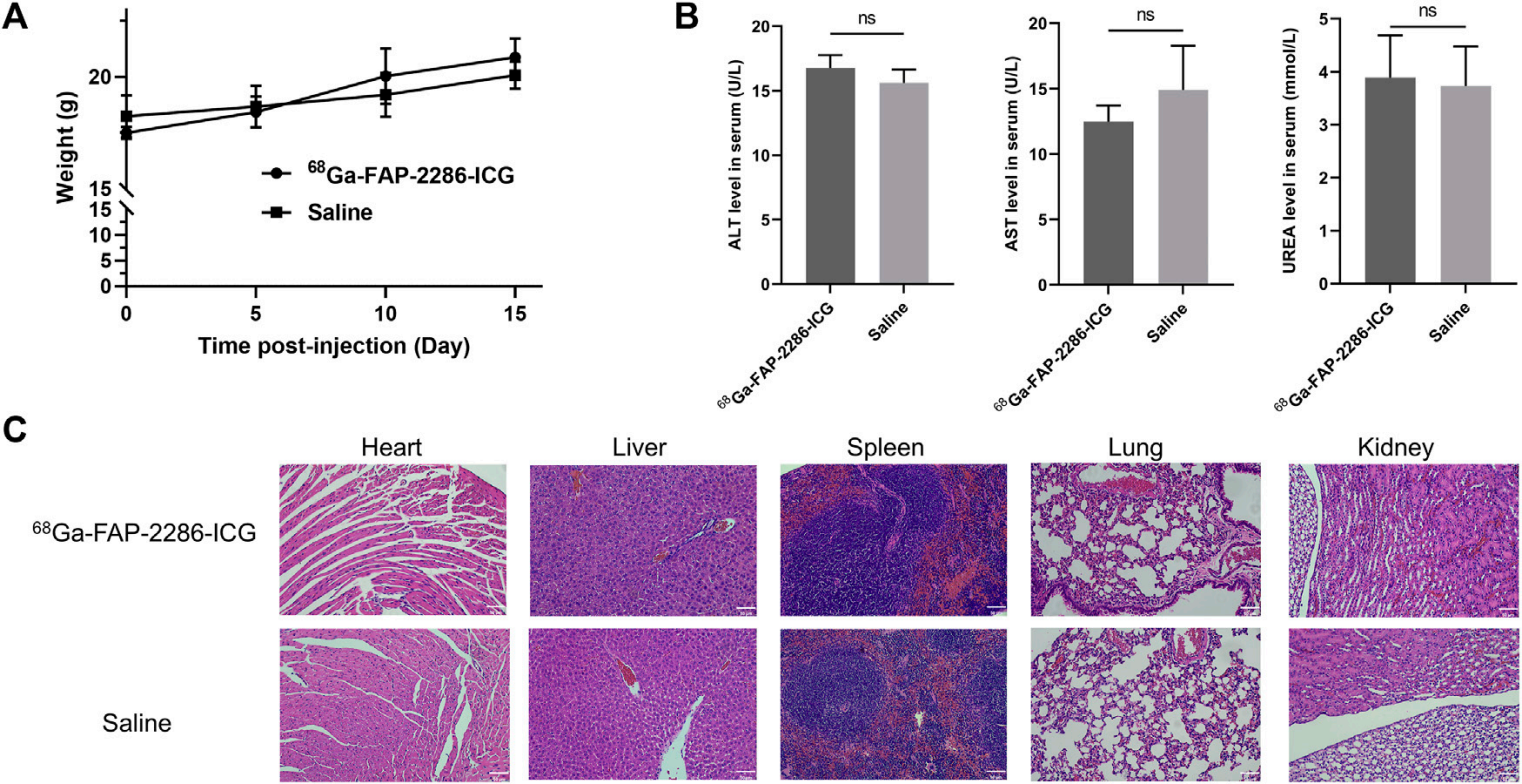
Toxicity study of [^68^Ga]Ga-FAP-2286-ICG. **(A)** Change in body weight of mice after injection of [^68^Ga]Ga-FAP-2286-ICG or saline; **(B)** ALT, AST, and UREA levels in mice 3 weeks after injection of [^68^Ga]Ga-FAP-2286-ICG or saline; **(C)** H&E staining of major organs (magnification × 200). Scale bar: 50 μm. n = 3 for the group treated with [^68^Ga]Ga-FAP-2286-ICG and the control group. ns: not statistically significant.

The toxicity of [^68^Ga]Ga-FAP-2286-ICG was assessed in BALB/c mice, and no evident signs of acute toxicity were observed during a 3-week period. After injection, there were no significant differences in weight fluctuation between the treatment and control groups (Figure 7A). The serum concentrations of aspartate aminotransferase (AST), alanine aminotransferase (ALT), and blood urea nitrogen (UREA) did not exhibit significant changes 3 weeks after injection, when compared to the control group (Figure 7B). No significant pathological abnormalities were observed in the major organs, such as the heart, liver, spleen, lung, and kidney (Figure 7C). These results imply that [^68^Ga]Ga-FAP-2286-ICG is safe and further translational research is warranted.

## 4 Discussion

This study designed a novel fluorescent probe targeting FAP using the high tumor retention cyclic peptide FAP-2286 and the FDA-approved fluorescent dye ICG. The preparation and radiolabeling process for [^68^Ga]Ga-FAP-2286-ICG was simple and achieved a high radiochemical yield and purity. This fast, simple, and robust radiolabeling process will help to support clinical studies further. Additionally, FAP-2286-ICG has identical excitation and emission wavelengths to ICG, obviating equipment replacement and enhancing prospects for routine clinical usage.

In recent years, fluorescent probes targeting FAP such as FTL-S-S0456 and HCFP have been pursued for cancer imaging (Xing et al., 2018; Mukkamala et al., 2022). It has demonstrated favorable tumor retention and good fluorescence imaging in subcutaneous xenograft models of various tumor types, including HNC. However, inherent limitations exist with single modality fluorescent imaging, including insufficient tissue penetrance. Building upon previous works, our study demonstrates several innovative aspects. First, FAP-2286 peptide was employed as the tumor-targeting ligand, whose high binding affinity and retention are advantageous for prolonged intraoperative fluorescence guidance. In contrast, probes such as HCFP exhibit faster clearance kinetics. Notably, we optimized the design of our PET/fluorescent probe specifically for head and neck cancers, considering the unique clinical needs of this patient population. Collectively, [^68^Ga]Ga-FAP-2286-ICG showed promising results in preclinical studies for precise localization and fluorescence-guided resection of HNC tumors. With further optimization, this technique could be translated to improve therapeutic management of cancers in the clinic.

Furthermore, integrating PET and intraoperative FGS by employing the same imaging agent for preoperative patient selection and surgical planning offers a cost-effective approach with signal congruence. This strategy reduces drug development costs and ensures consistent and accurate imaging results across modalities. This congruence allows for a seamless transition and correlation of preoperative imaging findings with intraoperative visualization, facilitating precise surgical planning and guidance. The integration of these techniques enhances the precision and efficacy of surgical interventions, leading to improved patient outcomes in oncological procedures.

Recent advancements in drug delivery protocols aim to enhance treatment adherence by simplifying dosing frequency (Baryakova et al., 2023). In this study, we demonstrated that a single-dose strategy provided up to 72 h of sustained tumor-specific imaging capabilities, with impressive tumor-to-background ratios in FI. Dual-modality probes offer substantial advantages in medical imaging by enabling multiple imaging modalities with a single-dose administration. The benefits encompass improved patient convenience, streamlined examination processes, simplified interpretation, and optimized treatment planning. The fluorescent signal in the tumor can be retained for a prolonged period, allowing for flexible operating time and an extended surgical window for the surgeon. This interval also ensures sufficient radionuclide decay (^68^Ga half-life: 68 min), which can significantly reduce or even eliminate radiation damage to the surgeon. However, further research and validation are essential to ascertain the feasibility and applicability of this approach in diverse clinical scenarios.

The precise excision of primary or metastatic tumors during HNC treatment significantly affects the prognoses of patients. Novel methodologies employing dual-labeled probes aid the surgeon by allowing for more streamlined preoperative planning via PET/CT or PET/MRI and accurate intraoperative navigation guidance. Van den Berg et al. (van den Berg et al., 2012). previously demonstrated the potential of adopting this dual labeled diagnosis procedure into a clinical workflow by demonstrating that multimodal ICG-[^99m^Tc]Tc-nanocolloids can visualize the sentinel lymph nodes of oral squamous cell cancer. However, the role of ICG accumulation in tumors was presumably due to the EPR effect of the tumor. The lack of tumor-specific characteristics of ICG limits its efficacy to guide surgical resection. Here, our work aptly confirmed the potential of the dual labeled diagnosis procedure [^68^Ga]Ga-FAP-2286-ICG PET/NIR to guide preoperative assessment and intraoperative navigation in HNCs. In parallel, the technique further improved the precise localization of tumors and reduced the dose of drug injected, benefiting from the targeting properties to the FAP. In this study, mice were injected with a dose of FAP-2286-ICG (0.158 mg/kg) equivalent to the human equivalent dose of 0.0128 mg/kg (Reagan-Shaw et al., 2008), which is well below the clinically safe dose of ICG (5 mg/kg).

While this proof-of-concept study demonstrates the potential of [^68^Ga]Ga-FAP-2286-ICG PET/NIR imaging for HNC diagnosis and image-guided surgery, there are some limitations of this study that must be acknowledged. The subcutaneous xenograft models using cancer cell lines may not fully capture the complexity of actual human tumors. Further validation in more clinically relevant orthotopic and genetically engineered mouse models is needed. Moreover, additional optimization of the PET/NIR imaging interval may further improve dual-modality visualization. Overall, this early-stage work shows promising translational potential but still requires more rigorous preclinical validation before progression to human studies. Interestingly, based on the PET/CT imaging results of the HNC model, we observed differences in probe uptake by xenografts from different cell lines, despite the fact that the 3 cell lines (FaDu, CAL27, and CNE2) do not inherently express FAP. We speculate that several factors may contribute to the variable tumor uptake of the FAP-targeted agent. Diversity in recruitment of FAP-positive CAFs into the tumor microenvironment could lead to differences in FAP availability and probe binding. Discrepancies in angiogenesis may also affect probe delivery and clearance. Furthermore, variations in proliferation rates and immunogenicity could indirectly impact stromal cell accumulation and the overall tumor milieu. Further investigations controlling for these factors will elucidate the mechanisms underlying heterogeneous uptake between models. In recent years, imaging techniques using the near-infrared window II region (NIR-II, 1,000–1,700 nm) have demonstrated much higher resolution and more accurate information on deep tissues than NIR-I imaging techniques. NIR-II imaging can improve the accuracy and efficiency of intraoperative precision tumor resection and is of great potential for tumor treatment. Notably, the NIR-I dyes ICG have recently been discovered to exhibit tail fluorescence in the NIR-II window. ICG or ICG-conjugated antibodies and peptides have been demonstrated to possess high-performance NIR-II imaging capabilities in both clinical and pre-clinical models (He et al., 2018; Hu et al., 2020; Cao et al., 2023). Theoretically, it is highly likely that FAP-2286-ICG has the capability to expand into NIR-II imaging. However, further validation is warranted to confirm its potential diagnostic performance in NIR-II imaging.

## 5 Conclusion

This study has developed a FAP-targeted bimodal imaging probe [^68^Ga]Ga-FAP-2286-ICG for precision imaging and image-guided surgery of HNCs. The probe was constructed with the clinically validated cyclic peptide FAP-2286, which has high tumor uptake and retention, and the FDA-approved ICG. Furthermore, we demonstrated that the probe exhibited excellent imaging performance, such as good tumor uptake and high tumor-to-background ratios in preclinical models of HNCs, with high clinical and translational potential. Therefore, these initial findings on targeted dual-modality PET/NIR image-guided surgery demonstrate its potential to advance oncological surgery and improve the survival outcomes of patients with HNCs.

## Data Availability

The original contributions presented in the study are included in the article/Supplementary Material, further inquiries can be directed to the corresponding author.
