# Supplementary material for: Development of a fibroblast activation protein-targeted PET/NIR dual-modality probe and its application in head and neck cancer
Source: Front Bioeng Biotechnol. 2023 Nov 3;11:1291824. doi: 10.3389/fbioe.2023.1291824 (PMC10654779; doi:10.3389/fbioe.2023.1291824)

Supplementary Material

# Supplementary Figures

**Supplementary Figure S1.** The mass spectrometry analysis confirmed the successful synthesis of FAP-2286-ICG with the molecular weight of 2312.91.


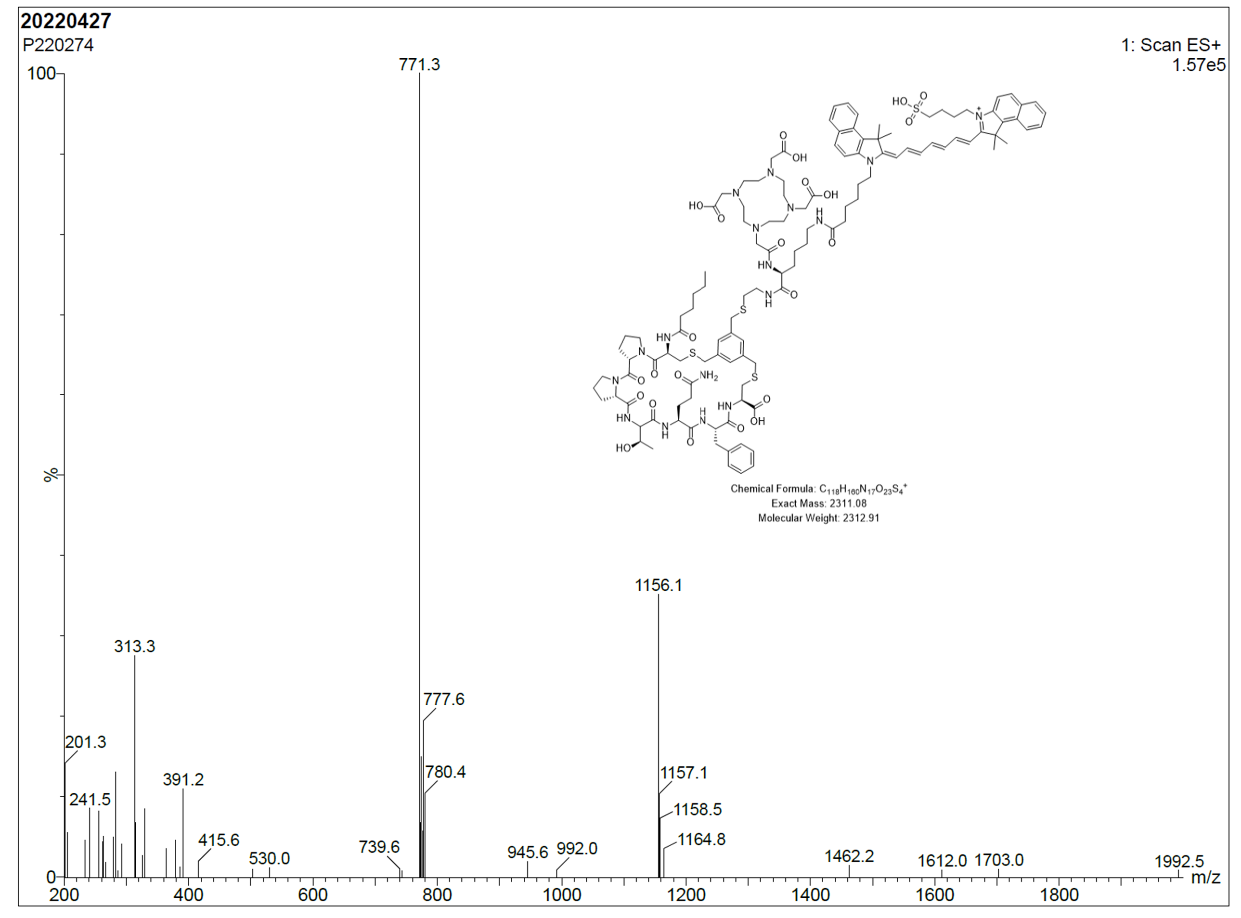


**Supplementary Figure S2.** The HPLC report of FAP-2286-ICG. The area percent calculation indicates that the chemical purity of FAP-2286-ICG is 99.19%.

**
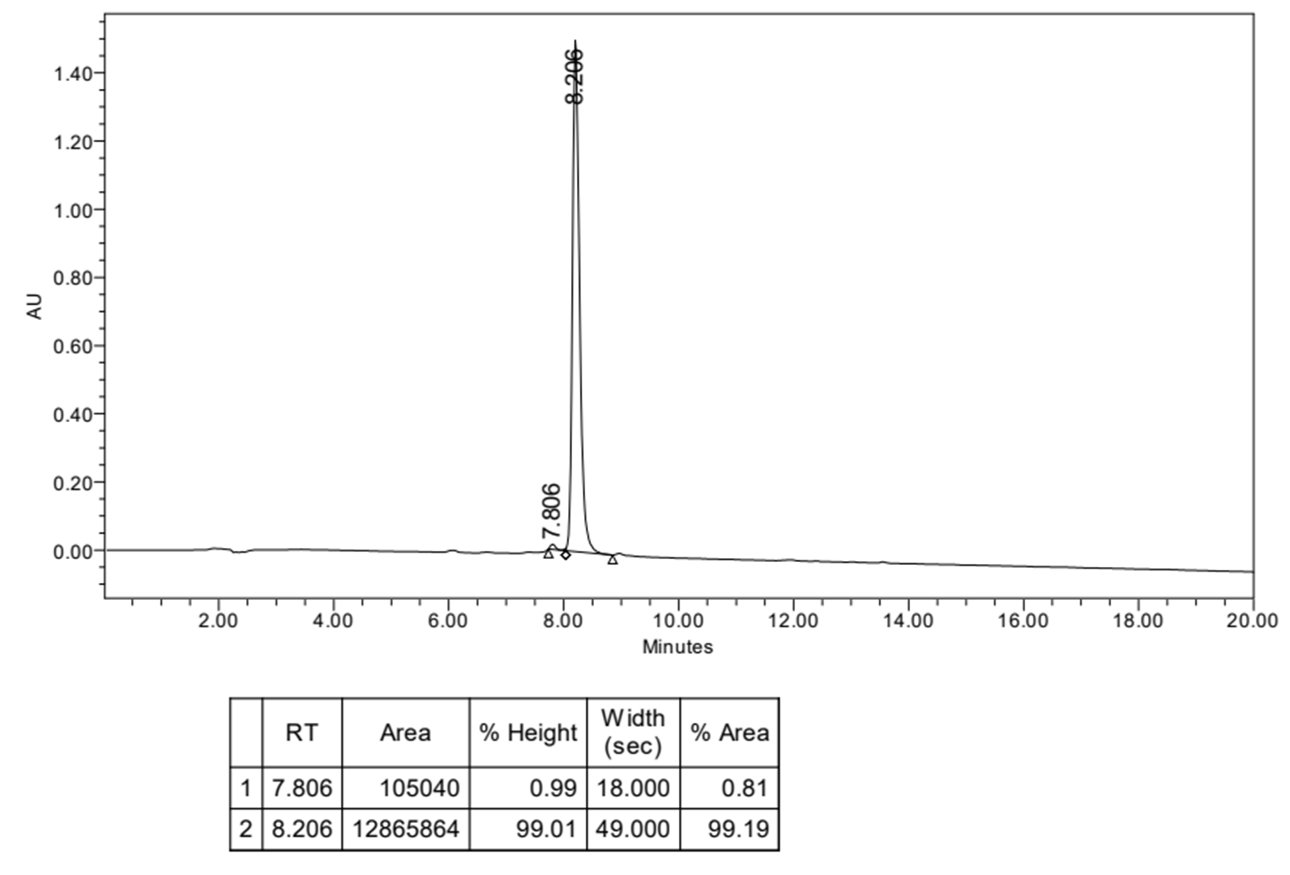
**

**Supplementary Figure S3.** In vivo NIR fluorescence imaging of FAP-2286-ICG in normal nude mice. A. Representative NIR fluorescence images of normal nude mice at 10 min after intravenous injection of 250 μL FAP-2286-ICG at varying concentrations (0-25 μM); B. Typical NIR images of normal nude mice at multiple time points after intravenous injection of 250 μL of FAP-2286-ICG at concentrations of 6.25, 12.5, and 25 μM.


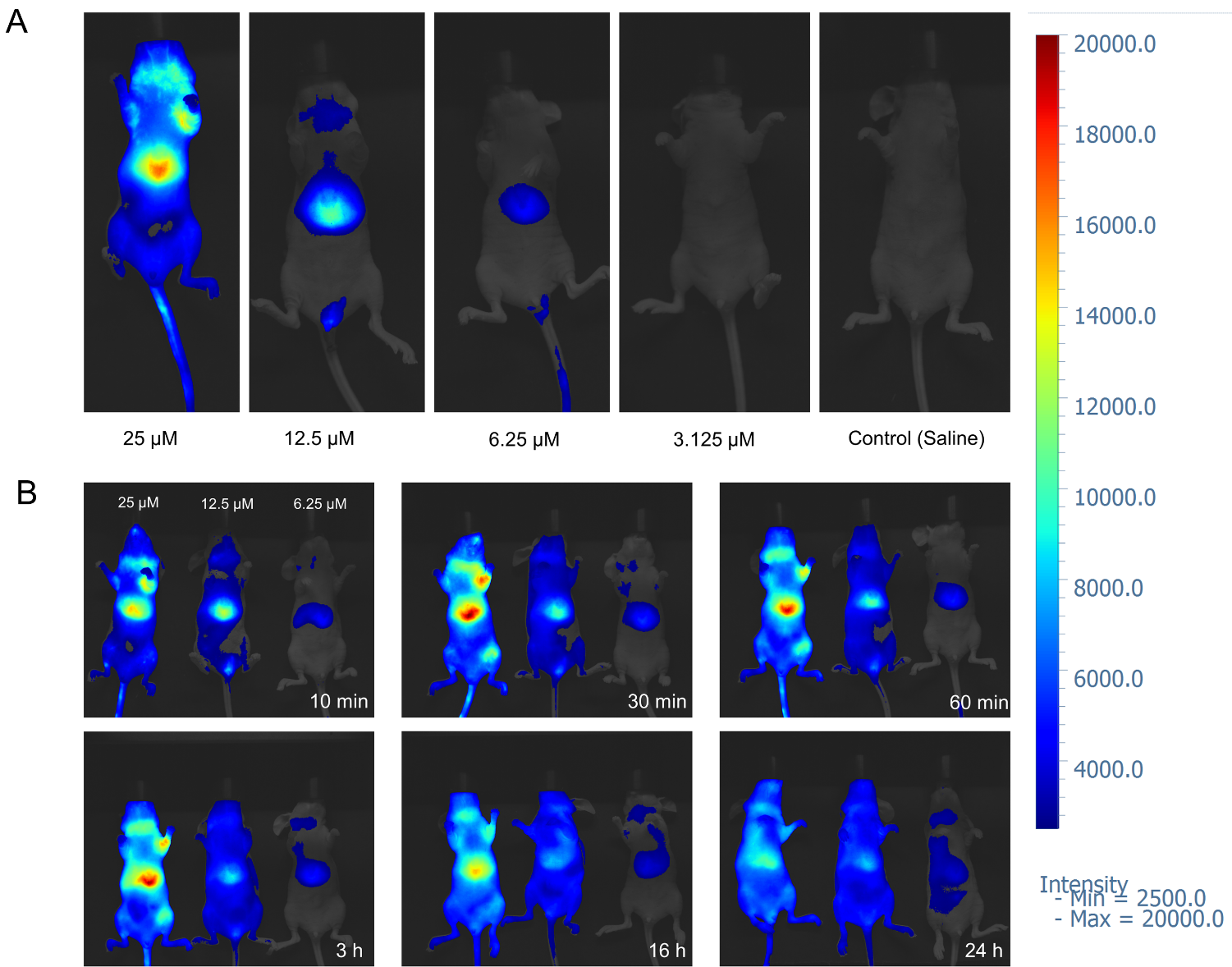

Supplement: Supplementary file 1 [file DataSheet1.docx]
